# Effect of COVID-19 infection related experiences on social behaviors when a state of emergency is declared: a cohort study

**DOI:** 10.1186/s12889-022-14864-5

**Published:** 2022-12-28

**Authors:** Takahiro Mori, Tomohisa Nagata, Kazunori Ikegami, Ayako Hino, Seiichiro Tateishi, Mayumi Tsuji, Shinya Matsuda, Yoshihisa Fujino, Koji Mori, for the CORoNaWork project, Akira Ogami, Hajime Ando, Hisashi Eguchi, Keiji Muramatsu, Kosuke Mafune, Makoto Okawara, Mami Kuwamura, Ryutaro Matsugaki, Tomohiro Ishimaru, Yu Igarashi

**Affiliations:** 1 Department of Occupational Health Practice and Management, Institute of Industrial Ecological Sciences, University of Occupational and Environmental Health, Japan, 1-1 Iseigaoka Yahatanishi-Ku, Kitakyushu, Fukuoka 807-8555 Japan; 2 Department of Work Systems and Health, Institute of Industrial Ecological Sciences, University of Occupational and Environmental Health, Japan, 1-1 Iseigaoka Yahatanishi-Ku, Kitakyushu, Fukuoka 807-8555 Japan; 3 Department of Mental Health, Institute of Industrial Ecological Sciences, University of Occupational and Environmental Health, Japan, 1-1 Iseigaoka Yahatanishi-Ku, Kitakyushu , Fukuoka 807-8555 Japan; 4 Disaster Occupational Health Center, Institute of Industrial Ecological Sciences, University of Occupational and Environmental Health, Japan, 1-1 Iseigaoka Yahatanishi-Ku, Kitakyushu , Fukuoka 807-8555 Japan; 5 Department of Environmental Health, School of Medicine, University of Occupational and Environmental Health, Japan, 1-1 Iseigaoka Yahatanishi-Ku, Kitakyushu, Fukuoka 807-8555 Japan; 6 Department of Preventive Medicine and Community Health, School of Medicine, University of Occupational and Environmental Health, Japan, 1-1 Iseigaoka Yahatanishi-Ku, Kitakyushu, Fukuoka 807-8555 Japan; 7 Department of Environmental Epidemiology, Institute of Industrial Ecological Sciences, University of Occupational and Environmental Health, Japan, 1-1 Iseigaoka Yahatanishi-Ku, Kitakyushu, Fukuoka 807-8555 Japan

**Keywords:** COVID-19, Self-restraint, Social behavior, Japan’s state of emergency

## Abstract

**Background:**

Restricting the movement of the public to gathering places and limiting close physical contact are effective measures against COVID-19 infection. In Japan, states of emergency have been declared in specific prefectures to reduce public movement and control COVID-19 transmission. We investigated how COVID-19 infection related experiences including people with a history of infection, people with a history of close contact, and people whose acquaintances have been infected, affected self-restraint from social behaviors during the second state of emergency in Japan.

**Methods:**

A prospective cohort study was conducted among workers aged 20–65 years using data from an internet survey. The baseline survey was conducted on December 22–25, 2020, and a follow-up survey was on February 18–19, 2021. There were 19,051 participants who completed both surveys and were included in the final analysis. We identified eight social behaviors: (1) eating out (4 people or fewer); (2) eating out (5 people or more); (3) gathering with friends and colleagues; (4) day trip; (5) overnight trip (excluding visiting home); (6) visiting home; (7) shopping for daily necessities; and (8) shopping for other than daily necessities. We set self-restraint regarding each social behavior after the second state of emergency was declared in January 2021 as the dependent variable, and COVID-19 infection related experiences as independent variables. Odds ratios were estimated using multilevel logistic regression analyses nested in the prefecture of residence.

**Results:**

Significant differences by COVID-19 infection related experiences were identified: compared to people without COVID-19 related experiences, people with a history of COVID-19 were less likely self-restraint from most social behaviors. People whose acquaintance had been diagnosed with COVID-19 were significantly more likely to refrain from most social behaviors. There was no significant difference in any social behaviors for people with a history of close contact only.

**Conclusion:**

To maximize the effect of a state of emergency, health authorities should disseminate information for each person in the target population, taking into account potential differences related to the infection related experiences.

## Introduction

The coronavirus disease 2019 (COVID-19) has been spreading worldwide since 2019. The known routes of COVID-19 infection include droplet infection, aerosol infection, and contact infection, so many infections occur in places where people gather or are in close physical contact [1].

One of effective control measures for COVID-19 infection is to reduce opportunities for people to go to places where people gather or have close physical contact with others [1, 2], and the most powerful measure is a restriction of behavior, the so-called lockdown. Lockdowns policies were taken in many countries, although there are differences in methods and degrees of severity. In Japan, the relatively less strict method is to declare a state of emergency and request the citizens to refrain from outing and social gathering. By the end of 2021, a total of four states of emergency have been declared due to the epidemic situation of COVID-19; the first April thru May 2020 against the first wave, the second January thru March 2021against the third wave arrived; the third April thru June 2021 against the fourth wave, and the fourth July thru September 2021 against the fifth wave [3]. The state of emergency is decided by each prefecture according to the infection status and the burden of medical institutions. Under the government's basic policy of refraining from outing and social gathering, specific measures differ slightly from prefecture to prefecture. In addition, even in prefectures without the state of emergency, each prefecture will consider the measures according to the infection status [4].

The effectiveness of such lockdown policies and states of emergency is likely to be influenced by how much the citizens actually refrain from social behaviors such as outing and gathering. Some studies have been conducted on sociodemographic factors that influence on social behaviors. Women, older people, highly educated people, and high-income earners are reported to more likely to refrain from social behaviors during a lockdown [5–14]. However, it has been reported that marital status, and whether they have an underlying disease are not associated with social behaviors during a lockdown [5, 6, 10, 11]. In Japan, reports noted that women and young people, people living with family, low-income earners, unemployed people, and those with an underlying disease are more likely to refrain from social behaviors during a state of emergency [15, 16], so some factors differ from the reports of other countries during a lockdown.

COVID-19 infection related experiences may also influence the effectiveness of lockdown policies and states of emergency. COVID-19 infection related experiences can be classified into (1) people with a history of infection, (2) people with a history of close contact, and (3) people with acquaintances who have been infected. It was reported people who thought they had had COVID-19 were more likely to think that they had some immunity to the virus and were less likely to adhere to social distancing measures [17]. Conversely, people with a history of close contact or acquaintances may think that they were likely to be infected and further strengthen the measures against infection and refrain from social behaviors, but these effects have not yet been investigated to our knowledge. This is probably because it is relatively easy to find infected people through testing such as real-time reverse transcription-polymerase chain reaction (RT-PCR), but it was difficult to identify close contacts unless systematically conducted by a specialized agency. In that respect, Japan has focused on cluster measures to prevent the spread of infection, and public health centers have conducted “active epidemiological surveys”. In this survey, the public health centers confirmed the activity before the onset of symptoms for each infected person, and determined the close contacts. Moreover, close contacts undergo an RT-PCR test to check for infection, so they could clearly recognize whether they were infected or close contacts [18].

Individual behavior is known to be influenced by the behavior of others, and they sometimes choose bad behavior [19], so it is necessary to identify those who do not respond to government policies and consider individual measures for them. We therefore investigated how each type of COVID-19 infection related experience affected self-restraint in social behaviors using the data in January 2021 when the second state of emergency was declared in Japan. If differences between COVID-19 infection related experiences are found, it is shown that considering measures against infection experiences in addition to sociodemographic factors in policy decisions during COVID-19 and other pandemic outbreaks is necessary.

## Methods

### Study design and participants

This prospective cohort study was undertaken by a research group from the University of Occupational and Environmental Health, Japan, called the Collaborative Online Research on Novel-coronavirus and Work study (CORoNaWork study). This survey was conducted as a self-administrated questionnaire by the internet survey company Cross Marketing Inc. (Tokyo, Japan). The baseline survey was conducted on December 22–25, 2020, and the follow-up survey was on February 18–19, 2021; both periods were during the third wave of the pandemic in Japan. Details of the study protocol have been previously reported [20]. Participants (*n* = 33,087) were aged 20–65 years and employed at the time of the baseline survey. Respondents to the CORoNaWork study were sampled taking into account region, occupation, and sex. After excluding 6,051 initial subjects who provided invalid responses, we ultimately included 27,036 in the database. Invalid responses were determined as follows: response time < 6 min, body weight < 30 kg, height < 140 cm, inconsistent answers to similar questions, and wrong answers to a question used solely to identify unreliable responses.

**Fig. 1:**
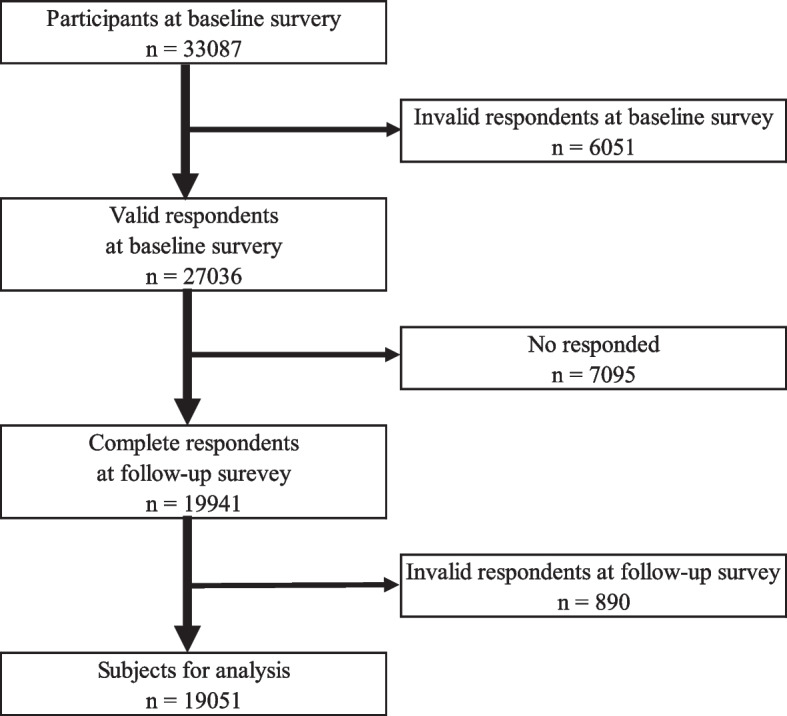
Flow chart of the study participants

These subjects were given a follow-up survey, and 19,941 people responded (74% follow-up rate). We excluded five participants who gave an inappropriate age and 885 participants who gave incorrect answers to a question that were only used to identify unreliable answers in the follow-up survey. Finally, 19,051 participants were included in the analysis. The flow diagram is shown in Fig. 1.

The present study was approved by the Ethics Committee of the University of Occupational and Environmental Health, Japan (Approval numbers: R2-079 and R3-006). Informed consent was obtained in the form of the website from all participants.

### Assessment of social behaviors

We identified eight social behaviors which the Japanese government requested for self-restraint [21]: (1) eating out (4 people or fewer); (2) eating out (5 people or more); (3) gathering with friends and colleagues; (4) day trip; (5) overnight trip (excluding visiting home); (6) visiting home; (7) shopping for daily necessities; and (8) shopping for other than daily necessities. We divided eating out into four people or fewer and five or more to identify any difference based on the number of people, particularly because the Japanese government has emphasized that drinking or dining with five or more people carries a particularly high risk of infection [21]. For each of the eight behaviors, we asked participants in the follow-up survey, “Has your self-restraint changed in response to the second state of emergency in January 2021?” Respondents chose one of the following five options: “increased a lot”, “increased a little”, “no change”, “decreased a little”, or “decreased a lot”. We created a binary variable by defining “decreased a little” or “decreased a lot” as having self-restraint behavior, and the others as not having self-restraint behavior.

### Assessment of the COVID-19 related experiences

In the baseline survey, we asked participants three questions about their COVID-19 infection related experiences: “Have you ever been infected with COVID-19?”, “Have you ever been in close contact with someone with COVID-19?”, and “Do you have an acquaintance who has been infected with COVID-19?” Respondents answered each question with “Yes” or “No”, and were classified into the four following types: people with a history of COVID-19; people without history of COVID-19 but with a history of close contact with cases of confirmed COVID-19 (hereinafter referred to as people with a history of close contact); people without a history of COVID-19 or close contact with cases of confirmed COVID-19 but who had an acquaintance who had been diagnosed with COVID-19 (hereinafter referred to as people whose acquaintance had been diagnosed); and people without history of COVID-19 or close contact with cases of confirmed COVID-19, and who did not have an acquaintance diagnosed with COVID-19 (hereinafter referred to as people without history of COVID-19 or close contact).

### Assessment of covariates

Covariates included demographics, socioeconomic factors, job type, underlying disease, and prefectures with and without the second state of emergency. Age was expressed as a continuous variable. Education was classified into three categories: junior high or high school, vocational school or college, and university or graduate school. Marital status was classified into three categories: married, divorced or widowed, and never married. Equivalent income was classified into four categories: < 2.50 million Japanese yen (JPY); 2.50–3.74 million JPY; 3.75–5.24 million JPY; and ≥ 5.25 million JPY (1 USD was equal to 106.78 JPY, using 2020 conversion rates) [22]. Job type was classified into three categories: mainly desk work, jobs mainly involving interpersonal communication, and mainly physical work. Regarding underlying disease, we asked the question, “Do you have any disease that requires regular visits to the hospital or treatment?” Participants selected one of the following: “I do not have such a disease,” “I have such a disease,” or “I have such a disease, but refrained from going to the hospital following the second state of emergency.” We rated the participants who answered, “I do not have such a disease” as “No” and the remaining two answers as “Yes.” There were 11 prefectures that had the second state of emergency, and 36 prefectures without this declaration.

### Statistical analyses

Multilevel logistic regression analyses were used to examine the association between COVID-19 infection related experience and self-restraint from social behaviors after the second state of emergency. An analysis was performed on each of the eight social behaviors. We estimated age-sex adjusted odds ratios (ORs) and multivariate adjusted ORs for each social behaviors using multilevel logistic regression analyses nested in the prefecture of residence to take account of regional differences in the infection status of COVID-19. The model included age, sex, education, marital status, equivalent income, job type, underlying disease, and prefectures with and without the second state of emergency. A p-value of less than 0.05 was considered statistically significant. All analyses were conducted using Stata Statistical Software (Release 16; StataCorp LLC, College Station, TX, USA).

## Results

Table 1 shows the participant characteristics for each category of COVID-19 infection related experience. There were 18,208 people without history of COVID-19 or close contact, 154 people with a history of COVID-19, 141 people with a history of close contact, and 1438 people whose acquaintance had been diagnosed. The mean age was youngest for people with a history of COVID-19. People without history of COVID-19 or close contact had a lower rate of being married than did other groups. In prefectures with the emergency declaration, there were fewer people without history of COVID-19 or close contact and more people with a history of COVID-19, with a history of close contact, and whose acquaintance was diagnosed than in prefectures without the declaration.

**Table 1:** Characteristics of participants by categories of COVID-19 infection related experience

	People without history of COVID-19 or close contact^a^	People with a history of COVID-19	People with a history of close contact^b^	People whose acquaintance had been diagnosed^c^
	*n* = 17,394	*n* = 141	*n* = 138	*n* = 1378
Age, mean (SD)	48.1 (10.1)	44.0 (11.5)	46.0 (10.6)	48.0 (10.2)
Sex, men	9787 (56.3%)	77 (54.6%)	85 (61.6%)	778 (56.5%)
Education				
Junior high or high school	4722 (27.1%)	34 (24.1%)	22 (15.9%)	335 (24.3%)
Vocational school or college	3988 (22.9%)	31 (22.0%)	37 (26.8%)	277 (20.1%)
University or graduate school	8684 (49.9%)	76 (53.9%)	79 (57.2%)	766 (55.6%)
Marital status				
Married	9695 (55.7%)	89 (63.1%)	84 (60.9%)	842 (61.1%)
Divorced or bereaved	1755 (10.1%)	13 (9.2%)	10 (7.2%)	156 (11.3%)
Never married	5944 (34.2%)	39 (27.7%)	44 (31.9%)	380 (27.6%)
Equivalent income				
< 2.50 million JPY	3797 (21.8%)	27 (19.1%)	16 (11.6%)	196 (14.2%)
2.50–3.74 million JPY	4360 (25.1%)	32 (22.7%)	30 (21.7%)	327 (23.7%)
3.75–5.24 million JPY	4605 (26.5%)	37 (26.2%)	38 (27.5%)	373 (27.1%)
≥ 5.25 million JPY	4632 (26.6%)	45 (31.9%)	54 (39.1%)	482 (35.0%)
Job type				
Mainly desk work	8991 (51.7%)	65 (46.1%)	61 (44.2%)	725 (52.6%)
Jobs mainly involving interpersonal communication	4206 (24.2%)	44 (31.2%)	44 (31.9%)	402 (29.2%)
Mainly physical work	4197 (24.1%)	32 (22.7%)	33 (23.9%)	251 (18.2%)
Underlying disease, Yes	6074 (34.9%)	63 (44.7%)	60 (43.5%)	539 (39.1%)
Prefectures with the second state of emergency, Yes	7419 (42.7%)	75 (53.2%)	91 (65.9%)	743 (53.9%)

*SD* Standard deviation, *JPY* Japanese yen

^a^People without history of COVID-19 or close contact with cases of confirmed COVID-19, and whose acquaintance was not diagnosed with COVID-19

^b^People with a history of close contact with cases of confirmed COVID-19 and without history of COVID-19

^c^People without a history of COVID-19 or close contact with confirmed cases of COVID-19 whose acquaintance had been diagnosed with COVID-19

Table 2 shows the association between COVID-19 infection related experiences and self-restraint from social behaviors during the second state of emergency. In multivariate analysis adjusted for age, sex, education, marital status, equivalent income, job type, underlying disease, and prefectures with and without the second state of emergency, people with a history of COVID-19 were less likely self-restraint from eating out (4 people or less) (OR = 0.48, 95% confidence interval (CI): 0.34–0.67), eating out (5 people or more) (OR = 0.48, 95% CI: 0.34–0.67), gathering with friends and colleagues (OR = 0.40, 95% CI: 0.29–0.57), day trip (OR = 0.52, 95% CI: 0.37–0.73), overnight trip (OR = 0.50, 95% CI: 0.35–0.70), and shopping other than daily necessities (OR = 0.68, 95% CI: 0.48–0.96) than people without history of COVID-19 or close contact, but there was no significant difference in visiting home and shopping for daily necessities. Conversely, people whose acquaintance had been diagnosed was significantly more likely self-restraint from eating out (4 people or fewer) (OR = 1.34, 95% CI: 1.19–1.51), eating out (5 people or more) (OR = 1.44, 95% CI: 1.28–1.62), gathering with friends and colleagues (OR = 1.49, 95% CI: 1.31–1.69), day trip (OR = 1.33, 95% CI: 1.18–1.49), overnight trip (OR = 1.33, 95% CI: 1.18–1.50), visiting home (OR = 1.19, 95% CI: 1.06–1.33), and shopping other than daily necessities (OR = 1.27, 95% CI: 1.14–1.42) than people without history of COVID-19 and close contact, but there was no significant difference for shopping for daily necessities. People with a history of close contact showed no significant difference in any of the social behaviors compared with people without history of COVID-19 and close contact.

**Table 2:** Association between COVID-19 infection related experience and self-restraint from social behaviors during the second state of emergency

	Self-restraint from social behaviors		Age-sex adjusted			Multivariate adjusted^a^		
	n	%	OR	95% CI	*P*	OR	95% CI	*P*
	(1) Eating out (4 people or fewer)							
People without history of COVID-19 or close contact^b^	9486	55	reference			reference		
People with a history of COVID-19	56	40	0.52	0.37–0.73	< 0.001	0.48	0.34–0.67	< 0.001
People with a history of close contact^c^	85	62	1.27	0.90–1.80	0.178	1.16	0.82–1.65	0.397
People whose acquaintance had been diagnosed^d^	880	64	1.43	1.27–1.60	< 0.001	1.34	1.19–1.51	< 0.001
	(2) eating out (5 people or more)							
People without history of COVID-19 or close contact^b^	10,061	58	reference			reference		
People with a history of COVID-19	60	43	0.53	0.38–0.74	< 0.001	0.48	0.34–0.67	< 0.001
People with a history of close contact^c^	89	64	1.29	0.91–1.84	0.154	1.15	0.81–1.65	0.431
People whose acquaintance had been diagnosed^d^	944	69	1.56	1.38–1.75	< 0.001	1.44	1.28–1.62	< 0.001
	(3) gathering with friends and colleagues							
People without history of COVID-19 or close contact^b^	11,391	65	reference			reference		
People with a history of COVID-19	65	46	0.46	0.33–0.64	< 0.001	0.40	0.29–0.57	< 0.001
People with a history of close contact^c^	91	66	1.03	0.72–1.47	0.858	0.91	0.63–1.30	0.605
People whose acquaintance had been diagnosed^d^	1041	76	1.62	1.42–1.84	< 0.001	1.49	1.31–1.69	< 0.001
	(4) day trip							
People without history of COVID-19 or close contact^b^	10,270	59	reference			reference		
People with a history of COVID-19	65	46	0.58	0.42–0.81	< 0.001	0.52	0.37–0.73	< 0.001
People with a history of close contact^c^	85	62	1.11	0.79–1.57	0.544	0.99	0.70–1.40	0.947
People whose acquaintance had been diagnosed^d^	930	67	1.43	1.27–1.61	< 0.001	1.33	1.18–1.49	< 0.001
	(5) overnight trip (excluding visiting home)							
People without history of COVID-19 or close contact^b^	10,504	60	reference			reference		10,504
People with a history of COVID-19	65	46	0.56	0.40–0.78	< 0.001	0.50	0.35–0.70	< 0.001
People with a history of close contact^c^	89	64	1.20	0.84–1.70	0.315	1.05	0.73–1.50	0.793
People whose acquaintance had been diagnosed^d^	951	69	1.45	1.29–1.64	< 0.001	1.33	1.18–1.50	< 0.001
	(6) visiting home							
People without history of COVID-19 or close contact^b^	8157	47	reference			reference		
People with a history of COVID-19	62	44	0.83	0.60–1.17	0.290	0.74	0.52–1.04	0.080
People with a history of close contact^c^	74	54	1.20	0.86–1.69	0.282	1.08	0.76–1.52	0.673
People whose acquaintance had been diagnosed^d^	749	54	1.29	1.15–1.44	< 0.001	1.19	1.06–1.33	< 0.001
	(7) shopping for daily necessities							
People without history of COVID-19 or close contact^b^	4746	27	reference			reference		
People with a history of COVID-19	51	36	1.44	1.02–2.04	0.040	1.39	0.98–1.96	0.068
People with a history of close contact^c^	44	32	1.24	0.87–1.79	0.238	1.20	0.84–1.73	0.319
People whose acquaintance had been diagnosed^d^	398	29	1.07	0.95–1.21	0.275	1.05	0.93–1.18	0.467
	(8) shopping for other than daily necessities							
People without history of COVID-19 or close contact^b^	7640	44	reference			reference		
People with a history of COVID-19	52	37	0.72	0.51–1.02	0.065	0.68	0.48–0.96	0.028
People with a history of close contact^c^	61	44	1.01	0.72–1.43	0.942	0.94	0.67–1.32	0.713
People whose acquaintance had been diagnosed^d^	707	51	1.33	1.19–1.49	< 0.001	1.27	1.14–1.42	< 0.001

*OR* Odds ratio, *CI* Confidence interval

^a^Adjusted for age, sex, education, marital status, equivalent income, job type, underlying disease, and prefectures with and without the second state of emergency

^b^People without history of COVID-19 or close contact with cases of confirmed COVID-19, and whose acquaintance was not diagnosed with COVID-19

^c^People with a history of close contact with cases of confirmed COVID-19 and without history of COVID-19

^d^People without a history of COVID-19 or close contact with confirmed cases of COVID-19 whose acquaintance had been diagnosed with COVID-19

## Discussion

We examined the association of COVID-19 infection related experiences with self-restraint from social behaviors during the second state of emergency. People with a history of COVID-19 reported significantly lower self-restraint from social behaviors than did people without a history of COVID-19 or close contact, except regarding shopping for daily necessities. Visiting home was also not significant but self-restraint tended to be less. People whose acquaintance had been diagnosed were significantly more likely to refrain from social behaviors except for shopping for daily necessities. The results of people with a history of COVID-19 and people whose acquaintance had been diagnosed were similar to what we had expected. It has been shown that risk perception for infection is involved in infection prevention behavior [23–25].

Risk perception for infection includes perceptions of the possibility and severity of infection [26]. The reason why people with a history of COVID-19 were less likely to refrain from social behaviors may result from their lower risk perception; they were less likely to be infected with COVID-19 again and less likely to become severe. The association between risk perception and preventive behavior was also reported in various countries during the 2009 H1N1 influenza epidemic [27–33]. It is consistent with the pervious study observed that people who believed they had had COVID-19 were more likely to report leaving home at early stage of COVID-19 in UK [17]. At the time of this survey, the number of infected people in Japan was less than 0.5% of the total population [34]. This infection rate was so low that infected people might have thought that they were unlucky even once infected, and infected persons often acquire neutralizing antibodies, so they might also have thought that re-infection was unlikely to occur [35, 36]. In addition, because past experiences have been reported to be less associated with concerns about voluntary risk behavior [29], a COVID-19 diagnosis might not lead to refraining from social behaviors that may have caused them to become infected in the past or increase their risk of infection. As for the perception of severity, if their infection was relatively mild or asymptomatic, they may have had perceived the severity of COVID-19 infection to be lesser. Accordingly, even when a state of emergency was declared, there was no change in self-restraint from social behaviors.

Conversely, people whose acquaintance had been diagnosed were considered to have increased risk perception. A survey of Japanese people showed that the risk to oneself is underestimated than the risk to society when comparing the degree to which one feels dangerous to oneself and the degree to one feels dangerous to society for the same infectious disease [37]. However, if a close acquaintance was infected or even became seriously ill, it is probable that the risk perception to oneself increased, leading to refraining from social behavior.

For people with a history of close contact, the results were different from our assumptions, and no significant difference was observed in any of social behaviors. The reasons for this result are not clear from this survey, but it is possible that there were both those who refrained from social behaviors and those who did not. In Japan, epidemiological surveys are conducted by health centers on each infected person, and close contacts are identified. After certification, close contacts undergo an RT-PCR test to check for infection and are quarantined at home for 14 days at the time of our survey, even if negative [18]. As mentioned above, people with a history of close contact felt close to the infection, so it is probable that they may have adopted the same behaviors as people whose acquaintance had been diagnosed. Conversely, it seems that there were a number of people who did not refrain from social behaviors. Some people may think that they have already been infected because of the possibility of false negatives even if the rt-PCR test is negative, or from the experience of being isolated at home for 14 days. In addition, many people are recognized as close contacts as a result of their family members living together being infected [38, 39], and often make decisions about social behaviors with their family members. These people therefore may have not refrained from social behaviors like people with a history of COVID-19.

Regarding the specific aspects of social behaviors, for shopping for daily necessities, neither people with a history of COVID-19 nor people whose acquaintance had been diagnosed showed a significant difference compared with people without history of COVID-19 and close contact. This may be because shopping for daily necessities is a daily activity necessary for daily life, unlike other social behaviors. Regarding eating out, similar results were seen in eating out with four people or fewer and with five or more, suggesting that they have a similar perception of the risk of infection.

Our results suggested that it is necessary to pay attention not only to sociodemographic factors that have already been investigated [5–16], but also to the COVID-19 infection related experiences. In particular, our finding that people whose acquaintance had been diagnosed were more likely to refrain from social behaviors suggested that in order to encourage people without COVID-19-related experiences to properly refrain from social behaviors, officials need to make these people feel more familiar to the risk of infection. Since it has been shown that the information effect is large [40], it is necessary to disseminate information that makes the infection more familiar according to the actual situation, and also that refraining from social behaviors is beneficial to society as a whole, including the national health care system. In addition, individual risk preference is known to be influenced by the decision-making of surrounding individuals [19], and it will be easier to make the choice to go out if many people go out when self-restraint is requested. It is therefore necessary to provide appropriate information to people with a history of COVID-19, such as the possibility of re-infection and the effects of new variants of SARS-CoV-2 [41].

There are several limitations to this study. First, we conducted an internet survey, which includes the possibility of selection bias. However, sampling was balanced by sex, occupation, and area of residence at the start of the study to reduce the potential for bias. Second, we classified the COVID-19 infection related experience using data from the baseline survey, so there may have been new people infected, close contacts, and people whose acquaintance had been diagnosed until January 2021 when the second state of emergency was declared. However, the ratio in each category implies that the number of newly applicable people was very small, and the period between the baseline survey and the second state of emergency was short, so we believe that it was unlikely to have a significant impact on the results. Third, we did not confirm the timing of the COVID-19 infection related experiences. For example, people who were recently infected may have thought that they were at lower risk of reinfection. There may be differences in risk perception depending on timing of the infection related experiences, but we have not taken this into account. Fourth, the outcome of interest in this survey was a decrease in self-restraint, the degree of social behaviors before the state of emergency and the specific degree of self-restraint during the declaration was not investigated. It may have been possible to clarify the impact of infection-related experiences on self-restraint in social behavior by confirming changes in social behaviors before and after the state of emergency, but we did not investigate the social behaviors before the declaration. In addition, those who had not done social behaviors at all before the second state of emergency was declared were included in the group who did not respond to self-restraint. However, the movement of people during the non-declaration period does not appear to have been significantly suppressed compared with before the COVID-19 epidemic [42]. This implies that there would have been few people in this category who did not go out at all during the non-declaration period.

## Conclusion

Our results show that the level of self-restraint from social behaviors due to a state of emergency differs depending on a subject’s experiences related to COVID-19 infection. When declaring a state of emergency in response to COVID-19 or other new infection pandemics in the future, in order to maximize the effect of the declaration, officials should consider measures that focus on the infection related experiences, such as disseminating information in a way that makes the infection feel more familiar.

## Data Availability

The datasets used and/or analysed during the current study are available from the corresponding author on reasonable request.

## Abbreviations

COVID-19: Coronavirus disease 2019
CORoNaWork: Collaborative Online Research on the Novel-coronavirus and Work
JPY: Japanese yen
OR: Odds ratio
